# Gut microbiome: a novel preventive and therapeutic target for prostatic disease

**DOI:** 10.3389/fcimb.2024.1431088

**Published:** 2024-07-29

**Authors:** Hongliang Cao, Difei Zhang, Pengyu Wang, Yishu Wang, Chengdong Shi, Hao Wu, Hao Du, Wenqiang Zhang, Zixuan Gou, Honglan Zhou, Song Wang

**Affiliations:** ^1^ Department of Urology II, The First Hospital of Jilin University, Changchun, China; ^2^ Key Laboratory of Pathobiology, Ministry of Education, Jilin University, Changchun, China; ^3^ Bethune First Clinical School of Medicine, The First Hospital of Jilin University, Changchun, China

**Keywords:** gut microbiome, prostate cancer, benign prostatic hyperplasia, prostatitis, therapy

## Abstract

The human gut microbiome (GM) impacts various physiological processes and can lead to pathological conditions and even carcinogenesis if homeostasis is disrupted. Recent studies have indicated a connection between the GM and prostatic disease. However, the underlying mechanisms are still unclear. This review aims to provide a summary of the existing information regarding the connection between the GM and various prostatic conditions such as chronic prostatitis/chronic pelvic pain syndrome (CP/CPPS), benign prostatic hyperplasia (BPH), and prostate cancer (PCa). Furthermore, the review aims to identify possible pathogenic mechanisms and suggest potential ways of targeting GM to prevent and treat prostatic disease. Due to the complexity of the mechanism between GM and prostatic diseases, additional research is required to comprehend the association between the two. This will lead to more effective treatment options for prostatic disease.

## 1 Introduction

Prostatic disease, such as noncancerous prostate conditions (NPC), which encompass prostatitis and benign prostate hyperplasia (BPH), along with prostate cancer (PCa), are common male urinary system issues globally. These conditions pose a significant economic burden to society (Fang et al., 2021). According to the NIH classification, prostatitis is classified into types I-IV (Krieger et al., 1999). The following analysis centers on Type III prostatitis, alternatively referred to as chronic prostatitis/chronic pelvic pain syndrome (CP/CPPS), making up the majority of cases at 90-95% and having the potential to impact males across all age groups. Signs of this condition may consist of discomfort in the lower abdomen, frequent urination, a sudden need to urinate, nighttime urination, problems with sexual function, and different mental health issues (Krieger et al., 2008; Schaeffer, 2008). As individuals age, the frequency of BPH increases, leading to worsening urinary symptoms like trouble starting urination, weak urine flow, urgent need to urinate, and more frequent urination. In severe cases, these symptoms may lead to urinary retention and secondary chronic renal dysfunction (Kahokehr and Gilling, 2014; Kim et al., 2016). PCa is the second most prevalent cancer in men globally and the most frequent in the male reproductive system. Individuals with PCa may show signs of urethral pressure, such as difficulties with urination, sudden inability to urinate, blood in the urine, and loss of bladder control. As the disease progresses, the tumor may invade the bones, resulting in bone pain, pathological fracture, anemia, spinal cord compression, and other poor prognoses (Nguyen-Nielsen and Borre, 2016; Teo et al., 2019; Sandhu et al., 2021). Despite the high frequency of prostatic disease and their significant effect on men’s well-being, the underlying causes are still not well understood (Schaeffer, 2008; Kahokehr and Gilling, 2014; Sandhu et al., 2021). This lack of understanding has resulted in inadequate prevention and prognosis of prostatic disease. Therefore, searching for new preventive therapeutic targets is necessary to improve the situation.

Various microorganisms, such as bacteria, archaea, fungi, viruses, and protozoa, are present in the intricate ecosystem of the human gut. These microorganisms are collectively referred to as gut microbiota. The gut microbiome (GM) refers to the surroundings, including the microbiota, any proteins or metabolites produced by them, their metagenome, and host proteins and metabolites present in this setting (Whiteside et al., 2015; Porter et al., 2018). The GM is essential in overseeing different physiological functions, including intestinal homeostasis, intestinal mucosal barrier function, inflammation, immune regulation, metabolic balance, and endocrine equilibrium in the body (Kuziel and Rakoff-Nahoum, 2022). Research has shown that an imbalance in the microbial species composition, known as dysbiosis, plays a role in the development of several diseases, such as Parkinson’s disease, Alzheimer’s disease, hypertension, atherosclerosis, obesity, diabetes mellitus, gestational diabetes mellitus, non-alcoholic fatty liver disease, inflammatory bowel disease, and colon cancer (Illiano et al., 2020). Additionally, the microbiota composition can affect the effectiveness and tolerability of disease treatments and medications (Badgeley et al., 2021). Furthermore, recent studies suggest that the microbiome, particularly that of the gut, may play a role in the development and progression of disease within the urinary tract, including prostatic disease, and as such, the ‘gut-prostate axis’ should be considered when treating patients (Jones-Freeman et al., 2021). To the end, we summarized research on the association between GM and prostatic disease, including possible pathogenic mechanisms and potential ways to target GM for prevention and treatment.

## 2 Observational and experimental evidence indicating a strong link between GM and prostatic disease

Recent developments in biological techniques, including high-throughput sequencing like 16S rRNA sequencing, transcriptomics, and metabolomics, offer a chance to examine the GM pattern and its relationship with the advancement of prostatic disease (Jones-Freeman et al., 2021). Recent observational studies show that the GM and metabolites of men with prostate disease are significantly different from those of healthy men. This indicates that specific intestinal microorganisms could play a role in the development, advancement, and modified treatment outcomes of prostate conditions. Additionally, several animal studies have reported similar findings. **Table 1** shows clinical studies and animal experiments related to prostate disease, showing notable variations in gut microbiota and metabolites between the experimental and control groups.

**Table 1:** Observational and experimental studies reveal the association between GM and prostatic disease.

Prostatic disease	First author/Year	Country	Subject	Sample size	Test method	Main outcomes	Reference
**CP/CPPS**	Daniel A Shoskes, 2016	America	human	50	16S rRNA gene sequencing	(1) Patients with CP/CPPS having decreased alpha diversity (p=0.001); (2) 3 taxa were overrepresented in cases and 12 were underrepresented, eg *Prevotella*.	(Shoskes et al., 2016)
	Shuai Wang, 2023	China	human	84	16S rRNA gene sequencing	(1) Alpha-diversity makes no difference, but Beta-diversity does; (2) three bacterial species were highly representative and seven bacterial species were low.	(Wang et al., 2023)
	Junsheng Liu, 2021	China	rat	12	16S rDNA sequencing, transcriptomic analysis, methyl C-capture sequencing,	(1) an altered structure of the GM; (2) 185 genes of intestinal epithelium associated with immune system, digestive system, metabolic process, changed; (3) 73,232 differentially methylated sites in the DNA of intestinal epithelium.	(Liu et al., 2021b)
**BPH**	Kentaro Takezawa, 2021	Japan	human	128	16S rRNA metagenomic analyses	(1) a higher proportion of *Bacillota* (*Firmicutes)* and Actinomycetota (*Actinobacteria)* and a lower proportion of *Bacteroidota* (*Bacteroidetes)*; (2) the *Firmicutes/Bacteroidetes* (F/B) ratio was significantly higher.	(Takezawa et al., 2021)
	Jinho An, 2023	Korea	rat	7	next-generation sequencing (NGS)	(1) an altered abundance of *Lactiplantibacillus, Flavonifractor, Acetatifactor, Oscillibacter, Pseudoflavonifractor, Intestinimonas*, and *Butyricimonas* genera; (2) finasteride treatment altered the abundance of *Barnesiella, Acetatifactor, Butyricimonas, Desulfovibrio, Anaerobacterium*, and *Robinsoniella* genera.	(An et al., 2023)
	Lu-Yao Li, 2022	China	rat	10	16S rDNA sequencing and liquid chromatography tandem mass spectrometry (LC-MS/MS)	(1) beta-diversity increased; (2) *Muribaculaceae, Turicibacteraceae, Turicibacter* and *Coprococcus* were decreased; (3) *Mollicutesand Prevotella* were increased; (4) the differential metabolites were enriched in steroid hormone biosynthesis, ovarian steroidogenesis, biosynthesis of unsaturated fatty acids and bile secretion; (5) *Prevotellaceae, Corynebacteriaceae, Turicibacteraceae, Bifidobacteriaceae* took part in metabolism.	(Li LY. et al., 2022)
**Pca**	Shaheen Alanee, 2019	USA	human	30	16 S rRNA gene sequencing	no significant GM difference found in two groups.	(Alanee et al., 2019)
	Weibo Zhong, 2022	China	Mouse and human	NA and 35	16 S rRNA gene sequencing	(1) the abundance of Pseudomonadota (*Proteobacteria)* was higher after antibiotic exposure; (2) intratumoral lipopolysaccharide (LPS) increased under the elevation of gut permeability, and the NF-κB-IL6-STAT3 axis was activated; (3) *Pseudomonadota* was enriched in patients with mPCa and was positively linked to plasma IL6 level, regional lymph node metastasis status, and distant metastasis status.	(Zhong et al., 2022)
	K S Smith, 2021	USA	human	16	16 S rRNA gene sequencing	(1) no differences in alpha diversity; (2) Beta-diversity metrics were different.	(Smith et al., 2021)
	Karen S Sfanos, 2018	USA	human	30	16 S rDNA gene sequencing	(1) a significant difference in alpha diversity; (2) compositional differences of men taking ATT, such as *Akkermansia muciniphila* and *Ruminococcaceae spp*; (3) an enriched gene pathways involved in steroid biosynthesis and steroid hormone biosynthesis of men taking oral ATT.	(Sfanos et al., 2018)
	David M Golombos, 2018	USA	human	20	next-generation sequencing (NGS)	(1) higher abundance of *Bacteriodes massiliensis*; (2) *Faecalibacterium prausnitzii* and *Eubacterium rectalie* had lower abundance; (3) relative gene, pathway, and enzyme abundance were different.	(Golombos et al., 2018)
	Joseph K M Li, 2021	China	human	86	16 S rRNA gene sequencing	(1) alpha/beta-diversity were significantly different after ADT; (2) *Ruminococcus gnavus* and *Bacteroides spp* had higher abundance while *Lachnospira* and *Roseburia* were reduced after ADT; (3) the F/B was lower after ADT; (4) biosynthesis of lipopolysaccharide (endotoxin) and propanoate and the energy cycle pathways were enriched after ADT.	(Li et al., 2021)
	Pin-Yu Huang, 2021	China	mouse	6	16 S rRNA gene sequencing	(1) *Akkermansiaceae, Bifidobacteriaceae* and *Enterococcaceae* were separately higher at different time in the progress of PCa; (2) bacterial associated with steroid biosynthesis and butirosin and neomycin biosynthesis were increased.	(Huang et al., 2021)
	Yufei Liu, 2020	China	human	21	16 S rRNA gene sequencing	(1) increased abundance of genus *Phascolarctobacterium* and *Ruminococcus* in CRPC; (2) bacterial gene pathways involved in terpenoids/polyketides metabolism and ether lipid metabolism were activated in CRPC.	(Liu and Jiang, 2020)
	Makoto Matsushita, 2021	Japan	human	152	16 S rRNA gene sequencing	(1) the relative abundances of *Rikenellaceae, Alistipes*, and *Lachnospira*, all SCFA-producing bacteria, were significantly increased in high-risk PCa;	(Matsushita et al., 2021)
	Michael A Liss, 2018	USA	human	105	16 S rRNA gene sequencing	(1) *Bacteroides* and *Streptococcus* species were higher in PCa; (2) Folate and argininewere the most significantly altered pathways.	(Liss et al., 2018)

GM, Gut microbiome; CP/CPPS, chronic prostatitis/chronic pelvic pain syndrome; BPH, benign prostatic hyperplasia; PCa, Prostate cancer; ATT, Androgen receptor axis-targeted therapies; ADT, Androgen deprivation therapy; SCFA, Short chain fatty acid.

### 2.1 The GM and CP/CPPS

A study comparing 25 individuals with CP/CPPS to 25 controls found that those with CP/CPPS had a lower diversity of gut microbiota, which formed distinct clusters compared to controls. Additionally, they had significantly fewer *Prevotella* bacteria, suggesting a possible biomarker for the condition (Shoskes et al., 2016). A different research study in China included 41 patients with CP/CPPS and 43 healthy controls, indicating notable variations in gut microbiota composition among the groups. The research developed an innovative diagnostic approach for CP/CPPS using microbiomes, showing potential for future treatment options and non-invasive diagnostic markers for patients with CP/CPPS (Wang et al., 2023). In addition, a comprehensive analysis examined CNP’s effects on the gut microbiota, gene expression, and DNA methylation in rats. The results showed strong associations between changes in gene expression, DNA methylation, and gut microbiota with various biological processes such as intestinal immunity, metabolism, and epithelial barrier function (Liu et al., 2021b). Observative research has noticed significant gut microbiota differences in CP/CPPS patients; however, there is no specific microbiota associated with CP/CPPS in different studies. Moreover, a large cohort is needed to prove this association.

### 2.2 The GM and BPH

A study involving 66 individuals with prostate enlargement (PE) and 62 controls found that the PE group had a higher percentage of *Bacillota* (*Firmicutes)* and Actinomycetota (*Actinobacteria)* and a lower percentage of *Bacteroidota (Bacteroidetes)*. In the PE group, the ratio of *Bacillota* to *Bacteroidota* was notably more excellent compared to the non-PE group (Takezawa et al., 2021). In addition, there is also a significant difference between BPH and controls in animal models. Certain gut microbiota levels have been noted to vary in rat models with BPH, including *Lactiplantibacillus, Flavonifractor, Acetatifactor, Oscillibacter, Pseudoflavonifractor, Intestinimonas, Butyricimonas, Muribaculaceae, Turicibacteraceae, Turicibacter*, and *Coprococcus*. *Lactiplantibacillus* and *Acetatifactor*, two types of microbiotas, were linked to supporting and preventing prostate cell apoptosis, respectively. These effects were reversed by finasteride, a medication often prescribed for BPH. Additionally, analysis of intestinal contents using LC-MS/MS showed that various metabolites linked to the production of steroid hormones, ovarian steroid synthesis, creation of unsaturated fatty acids, and release of bile were primarily related to cellular functions, processing environmental information, metabolism, and organismal systems, potentially linked to *Prevotellaceae, Corynebacteriaceae, Turicibacteraceae*, and *Bifidobacteriaceae* (Li LY. et al., 2022; An et al., 2023). These changes suggest their potential utility in diagnosing, preventing, and treating BPH.

### 2.3 The GM, androgen deprivation therapy, and PCa

Research examined the gut microbiota makeup and abundance, pinpointed specific metabolites and metabolic routes linked to PCa, and subsequently created a microbiome risk assessment for the disease. Research by K S Smith found that beta-diversity metrics significantly differed in PCa cases (Smith et al., 2021). A pilot study comparing men with BPH or clinically localized PCa at intermediate or high risk found that those with PCa had a more significant presence of *Phocaeicola massiliensis* (*Bacteroides massiliensis)* (Matsushita et al., 2021). Moreover, some specific microbiotas are abnormal in patients with PCa, such as *Bacteroides*, *Streptococcus* species (Liss et al., 2018), *Akkermansiaceae, Bifidobacteriaceae*, and *Enterococcaceae* (Huang et al., 2021). In addition, Weibo Zhong et al. discovered an abundance of *Pseudomonadota (Proteobacteria)* in individuals with metastatic prostate cancer (mPCa), which showed a positive association with plasma interleukin-6 (IL-6) levels, regional lymph node metastasis, and distant metastasis status (Zhong et al., 2022). Furthermore, it was found that the levels of *Rikenellaceae*, *Alistipes*, and *Lachnospira*, which are all bacteria that produce short-chain fatty acids(SCFAs), were notably higher in the high-risk group (Golombos et al., 2018). Apart from the microbiota difference, a discrepancy in metabolites derived from GM, such as folate and arginine, has also been found (Liss et al., 2018). Nonetheless, a future investigation discovered that examination of the bacterial classifications in the stool samples did not show any grouping corresponding to benign or malignant prostate biopsies (Alanee et al., 2019). Overall, while most studies indicate a notable distinction between PCa and men in good health, additional data is required to support these results.

Androgen deprivation therapy (ADT), using medications such as bicalutamide, enzalutamide, and abiraterone acetate, or surgical castration, is the mainstay of conventional care for locally advanced PCa or mPCa. Following a period of ADT, all individuals with PCa will eventually develop castration-resistant prostate cancer (CRPC), a fatal phase of the disease (Desai et al., 2021). Recent studies have suggested that ADT may interact with the GM and change the composition and abundance of specific microbiota, resulting in a shorter period in the process of CRPC. Following ADT, there was a notable change in the alpha/beta-diversity, with variations observed in *Mediterraneibacter gnavus (Ruminococcus gnavus)*, *Bacteroides*, *Lachnospira, Roseburia* (Li et al., 2021), *Akkermansia muciniphila* and *Oscillospiraceae (Ruminococcaceae) (* Sfanos et al., 2018 *).*, *Phascolarctobacterium* and *Ruminococcus* (Liu and Jiang, 2020). Bacterial gene pathways in fecal microbiota play a role in lipopolysaccharide (endotoxin), propanoate, terpenoids/polyketides metabolism, lipid metabolism, and steroid hormone biosynthesis, as shown in functional analyses (Sfanos et al., 2018; Liu and Jiang, 2020; Li et al., 2021).

Apart from epidemiological evidence, results from Mendelian randomization studies also suggested a causal link between gut microbiota and prostatic disease, which is an approach that employs single nucleotide polymorphisms (SNPs) as instrumental variables (IVs) to assess causal relationships, similar to randomized controlled trials (Xia et al., 2023; Xie and Hu, 2023; Shen et al., 2024).

## 3 The GM influences prostatic disease through multiple different potential mechanisms

**Figure 1:**
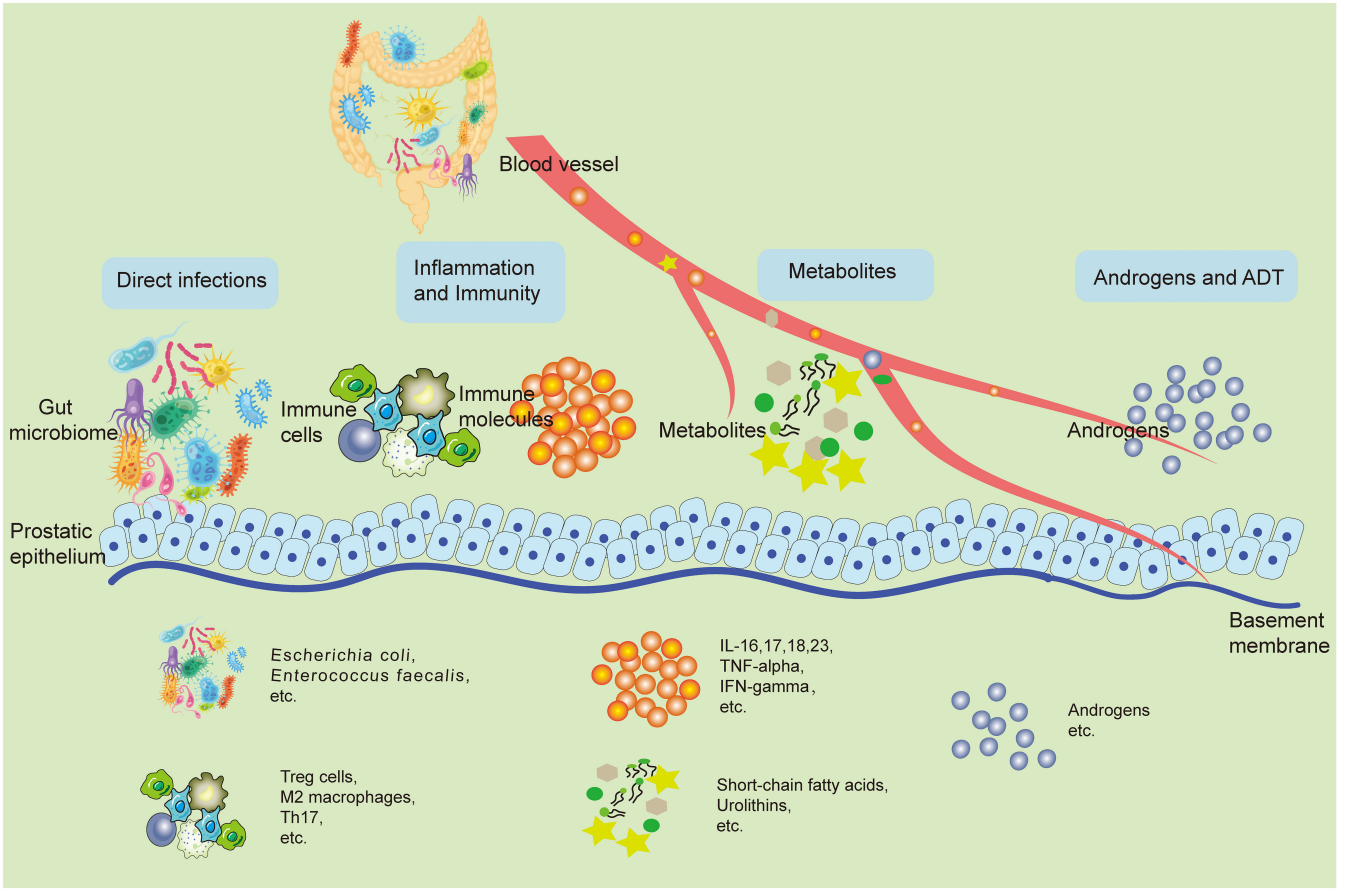
The potential mechanisms involved with the GM and its impact on prostatic disease pathogenesis. The GM can shadow prostate health in both direct and indirect ways. Bacteria from the gut can retroactively infect the prostate through the urethra and directly cause pathological states of the prostate. Changes in the local immune inflammatory state of the intestine can spread inflammatory factors and other immune molecules to the prostate through the bloodstream, causing changes in the local immune inflammatory environment of the prostate, leading to prostate disease. Metabolites from the gut microbiota like SCFAs, urolithins can also travel through the blood stream to the prostate, play a variety of bioactive functions, and directly lead to the activation of a variety of pathologic mechanisms of prostate cells. In addition, some gut microbiotas can change the synthesis and breakdown of androgens in the body, which directly affects the development of prostatic hyperplastic diseases, especially PCa. Accordingly, ADT treatment is also regulated by the GM. GM, Gut microbiome; SCFA, Short-chain fatty acids; PCa, Prostate cancer; ADT, Androgen deprivation therapy.

Although current observational studies have pointed to changes in the GM of patients with prostatic disease, and these characteristic differences have the potential to be targets for prevention and treatment, the exact mechanisms of their influence still need to be comprehensively clear. This part outlines the possible underlying principles associated with GM and how it affects the development of prostatic disease. This impact could be due to direct infections in the prostate caused by a known microbial cause, as well as indirect effects like immune system regulation, changes in metabolism, increased androgen activity, and effects on ADT. In many cases, frequent interactions with the GM, both direct and indirect, are in play (**Figure 1**).

### 3.1 Distant dissemination of gut pathogens

Experts and medical professionals acknowledge the harmful presence of bacteria in urinary tract infections (UTIs), which could play a role in causing prostate inflammation and prostatic disease development (Wagenlehner et al., 2014). Intestinal bacteria can enter the prostate through the urinary tract or travel through the bloodstream, resulting in infections that contribute to inflammatory conditions like bacterial prostatitis and chronic bacterial prostatitis, ultimately increasing the risk of BPH and PCa (Cai et al., 2019; Jin et al., 2024). Historically, urine bacteriological culture has been utilized for the purpose of isolating and identifying pathogens responsible for UTIs, including aerobic, rapidly multiplying organisms like *Escherichia coli* (80%) and *Enterococcus faecalis*, which primarily come from the intestinal tract (Ronald, 2003). Furthermore, various research has found and recognized bacteria in prostate tissues (Miyake et al., 2022). All of this evidence suggests that gut bacteria may cause pathological states of the prostate by directly infecting and interacting with the prostate.

### 3.2 Inflammation and immune dysregulation

The digestive system is not just important for breaking down food and taking in nutrients, it is also the primary immune system in the body, containing 60-80% of the body’s immune cells and structures that help regulate the immune system when faced with bacteria (Zhou et al., 2021). Dysbiosis can lead to the production of inflammatory cytokines like IL-17, IL-23, TNF-alpha, and IFN-gamma by the gut microbiota, which can then travel through the bloodstream to other parts of the body, such as the prostate, causing systemic inflammation. This inflammation can indirectly alter the local environment of the prostate, affecting immune molecules and cells, and potentially contributing to the development or progression of diseases (Porter et al., 2018; Russo et al., 2023).

In addition, SCFAs produced through the breakdown of carbohydrates by gut bacteria are vital in controlling the body’s functions and are seen as a key group of compounds responsible for this impact. Acetate, propionate, and butyrate play important roles in a variety of biological functions (Duan et al., 2023). New studies have uncovered the important roles they play in immune and inflammatory reactions. Butyrate inhibits the formation of interferon-gamma (IFN-γ) producing cells and enhances the formation of regulatory T (Treg) cells, as an example. Propionate hinders the start of a Th2 immune reaction by dendritic cells. SCFAs notably inhibit the polarization of M2 macrophages, highlighting their immunomodulatory characteristics and therapeutic potential. Furthermore, an imbalance in gut bacteria resulting in changes in SCFA production has been linked to the advancement of prostate diseases. SCFAs induce autophagy in cancer cells and stimulate M2 polarization in macrophages, hastening tumor progression. Moreover, SCFAs boost the activation of hypoxia-inducible factor 1 (HIF-1) through inhibition of histone deacetylase, leading to elevated synthesis of antimicrobial agents and enhanced macrophage-driven eradication of pathogens. This emphasizes the ability of SCFAs to fight against microbes and their importance in protecting the host. SCFAs are linked to the production of IL-6 and IL-18 in the prostate, and the balance of gut bacteria can influence the inflammatory environment in the prostate gland (Ratajczak et al., 2023). In a laboratory setting, SCFAs increased the movement and penetration of PCa cells by stimulating autophagy through TLR3 activation, subsequently triggering NF-κB and MAPK pathways. Concurrently, the autophagy process in PCa cells led to an increased secretion of chemokine CCL20, which could alter the tumor microenvironment by attracting additional macrophage infiltration and converting them into M2 type, ultimately enhancing the invasiveness of PCa cells. Additionally, among 362 patients with PCa, there was a positive association between the expression of CCL20 in prostate tissue and Gleason score, pre-surgery PSA levels, and invasion of neural/seminal vesicles. There was an inverse relationship with post-surgery biochemical recurrence-free survival (Liu et al., 2023). Moreover, a group created a mouse model of experimental autoimmune prostatitis (EAP) through subcutaneous immunization and found an imbalance in the frequency of Th17/Treg cells. Levels of propionic acid were lower in EAP mice than in control mice. Supplementing with propionic acid decreased susceptibility to EAP and restored the balance of Th17/Treg cell differentiation both *in vivo* and *in vitro*. Additionally, the impact of propionic acid on Th17 and Treg cells was assessed, including the regulation of SCFA receptor G-protein-coupled receptor (GPCR) 43 and intracellular histone deacetylase 6 (Du et al., 2022).

Matsushita et al. utilized *Pten*-knockout mice with prostate-specific characteristics to serve as a model for PCa and explored the cause of inflammatory cancer growth induced by a high-fat diet (HFD) and the role of the gut microbiome. In HFD mice with large prostate tumors, the levels of histamine and the expression of *Hdc* gene, which is responsible for histamine production, were increased, leading to an increase in mast cells surrounding the tumor foci. Fexofenadine administration, an H1 receptor antagonist, inhibited tumor growth in mice fed a HFD by decreasing myeloid-derived suppressor cells and inhibiting IL6/STAT3 signaling. Consuming a HFD led to an imbalance in gut bacteria, causing an increase in levels of lipopolysaccharide (LPS) in the bloodstream. PCa showed increased Hdc expression following intraperitoneal injection of LPS. Blocking the activation of LPS/Toll-like receptor 4 pathway reduced the growth of tumors induced by high-fat diet. In total prostatectomy specimens of severely obese patients, there was a rise in the quantity of mast cells surrounding the cancer foci (Matsushita et al., 2022b).

Immune elimination and immune escape are hallmarks of cancer; both can be partly bacteria-dependent in shaping immunity by mediating host immunomodulation. In addition, host immunity regulates the microbiome by altering bacteria-associated signaling to influence tumor surveillance. Cancer immunotherapy, including immune checkpoint blockade (ICB), appears to have heterogeneous therapeutic effects in different individuals, partially attributed to the microbiota. Thus, the microbiome signature can predict clinical outcomes, prognosis, and immunotherapy responses (Zhou et al., 2021).

### 3.3 Metabolites derived from the GM

Host metabolism is controlled by the gut microbiota. In recent years, it has been indicated that metabolites derived from GM play a vitally crucial role in gut homeostasis and could be transported to other body sites, including the prostate, exerting various functions. In addition to the high immune activity as mentioned in 3.1, SCFAs has many other biological activities involved in regulating prostate health. In a research study with 183 elderly males (103 with BPH and 80 without), researchers examined the types and amounts of SCFA in stool samples. The study revealed that patients with BPH had notably elevated levels of branched SCFAs, such as isobutyric acid and isovaleric acid (Ratajczak et al., 2021). In a research study, it was discovered that the amount of G protein-coupled estrogen receptor (GPER) expression, known for its ability to prevent prostate hyperplasia, was notably reduced in cases of prostate enlargement caused by ulcerative colitis (UC). Sodium butyrate could be up-regulated in the prostate when treated with sodium butyrate and increase the expression of GPER, which shows that SCFA could be a target for BPH (Dong et al., 2022). Furthermore, there was a decrease in propionic acid levels in the EAP mouse model when compared to controls. The addition of propionic acid supplementation decreased susceptibility to EAP, indicating that propionic acid could potentially serve as a protective factor for prostate health (Du et al., 2022). In addition, SCFAs may serve as intermediaries connecting dysbiosis of the microbiota in CRPC and the advancement of PCa. By using a transgenic TRAMP mouse model, *in vitro* PCa cell transwell, and macrophage recruitment assays, Yufei Liu et al. discovered that transferring fecal microbiota from patients with CRPC to TRAMP mice increased levels of SCFAs-producing gut bacteria like *Ruminococcus, Alistipes*, and *Phascolarctobacterium*, resulting in higher levels of gut SCFAs (acetate and butyrate). Supplementation with CRPC FMT or SCFA notably hastened the progression of PCa in mice. In a laboratory setting, SCFAs increased the movement and penetration of PCa cells by stimulating autophagy through TLR3, subsequently activating NF-κB and MAPK pathways (Liu et al., 2023). Makoto Matsushita’s study revealed that there is a higher presence of SCFA-producing bacteria like *Alistipes* in the gut microbiome of individuals with advanced PCa, leading to elevated levels of insulin-like growth factor 1 (IGF-1) in the prostate. IGF-1 has the ability to speed up the growth of PCa by activating phosphoinositide 3-kinase and mitogen-activated protein kinase, as well as enhancing sensitivity to sex hormones (Matsushita et al., 2022a). One review also noted the importance of SCFAs in maintaining cell balance by influencing histone deacetylases (HDACs), which impacts cell adhesion, immune cell recruitment, cytokine release, chemotaxis, and apoptosis (Mirzaei et al., 2021). Hence, changing the composition of gut bacteria to affect SCFA levels may be a viable option for preventing or treating prostatic disease.

Urolithins (Uros), which are derived from the metabolism of polyphenols, ellagitannins, and ellagic acid by gut microbiota, are able to be absorbed by the body. Iwona et al. found that Uros inhibited proliferation and induced apoptosis of LNCaP PCa cells (Stanisławska et al., 2019; García-Villalba et al., 2022). Combining bicalutamide with UroA and UroB resulted in an increased anti-proliferative impact. UroA and UroC reduced the secretion of prostate-specific antigen (PSA) induced by DHT (Stanisławska et al., 2018). Pioneering studies indicate that urolithins may play a role in the health benefits associated with consuming foods high in ellagitannins, such as pomegranates, walnuts, and strawberries. Uros and their associated metabolites that have been conjugated (glucuronides, sulfates, etc.) Various Uros can be found in a variety of human fluids and tissues, including the prostate (García-Villalba et al., 2022). This result indicated their potential use in complementary therapy of prostatic disease.

### 3.4 Androgens and ADT

Androgens are essential for the development and maintenance of healthy prostate cells, as well as hormone-responsive prostate cancer. ADT is a standard treatment for proliferative prostatic disease, including BPH and particularly PCa (Cui and Zhang, 2013). Nevertheless, through ADT, mPCa will ultimately become independent of androgens, leading to lethal CRPC, suggesting the existence of another origin of androgen (Zhang et al., 2022). Additionally, the GM can synthesize or decompose androgens, thereby affecting at least part of the systemic levels of the androgen, which could impact the effectiveness of ADT and lead to the emergence of CRPC. A study conducted in Japan included 54 male participants and identified certain bacteria from the *Bacillota* (*Clostridiales* etc.) were found to be increased in the PCa patients with serum high-testosterone expression, suggesting the GM may affect testosterone metabolism in older men (Matsushita et al., 2022c). Pernigoni et al. discovered that ADT in mice and humans supports the growth of specific beneficial bacteria, leading to the development of resistance to castration in mice. The GM in mice and CRPC patients was found to have an abundance of species that can transform androgen precursors into active androgens. Antibiotic treatment delayed the development of castration resistance in immunodeficient mice by eliminating the gut microbiota. Treatment from CRPC mice and patients made mice with PCa resistant to castration. Conversely, the progression of tumors was managed through the use of fecal microbiota transplantation (FMT) from patients with hormone-sensitive prostate cancer (HSPCa) and the administration of *Leyella stercorea* (*Prevotella stercorea)* (Fenner, 2021; Gut microbiota drive androgen resistance in prostate cancer, 2021; Pernigoni et al., 2021). *Clostridium scindens*, part of the intestinal microbiome, has the ability to transform cortisol into 11β-hydroxy androstenedione (11β-OHA), a strong precursor of androgens. Studies indicated that cortisol byproducts from *Clostridium scindens*-conditioned medium stimulated growth and increased movement of androgen-responsive PCa cells (LNCaP). Additionally, cells exposed to these compounds showed activated androgen receptor (AR) and induced AR-controlled genes (Bui et al., 2023). Lindsey K Ly et al. also found that certain bacteria, such as *Clostridium scindens*, could convert androgen precursors from the adrenal gland into potent androgens (Ly et al., 2020; Bui et al., 2023). In summary, these findings emphasize the importance of the GM in promoting CRPC tumor growth through stimulating androgen production and reveal a potential fecal bacterial pattern in patients that could serve as a useful indicator for identifying PCa patients who could potentially benefit from microbiome-focused treatment.

*Thauera* sp. strain GDN1 is an atypical beta proteobacterium, with the ability to break down androgen in both aerobic and anaerobic environments. Hsiao et al. gave C57BL/6 mice strain GDN1 orally, leading to a decrease of about 50% in serum androgen levels. The researchers hypothesized that it inhibited the reuse of androgens via enterohepatic circulation (Hsiao et al., 2023). Additionally, gut microbes that break down medications prescribed for androgen deprivation therapy may impact the effectiveness of the treatment (Terrisse et al., 2022b). The data suggest that androgen-degrading intestinal bacteria could be effective probiotics for treating hyperandrogenism in alternative therapy.

Conversely, oral drugs used for ADT like bicalutamide, enzalutamide, and abiraterone acetate may be associated with alterations in the gut microbiota, potentially leading to an increase in species known to influence response to anti-PD-1 immunotherapy, such as *Akkermansia muciniphila* and *Oscillospiraceae* spp. Additionally, a more detailed depiction of bacterial genetic pathways related to the production of steroids and steroid hormones was identified within the fecal microbiome (Sfanos et al., 2018). In research involving 23 individuals with PCa, it was found that the diversity of gut bacteria decreased notably after 24 weeks of ADT, with significant alterations in the levels of *Pseudomonadota, Gammaproteobacteria, Pseudomonadales, Pseudomonadota (Pseudomonas)*, and concentrations (Kure et al., 2023). ADT can reduce the population of *Corynebacterium* spp. that utilize androgens. In patients with PCa, oral Abiraterone acetate enhances the presence of the beneficial bacteria *Akkermansia muciniphila*. Additional research shows that Abiraterone acetate is broken down by bacteria in a laboratory setting, with specific components affecting growth in a way that could influence how patients with castrate-resistant conditions respond to treatment (Daisley et al., 2020). ADT administration in mice with PCa led to an increase in the number of cells in the thymus and the production of cells. Furthermore, the effectiveness of ADT was diminished by the depletion of the GM caused by oral antibiotics. PCa reduced the relative abundance of *Akkermansia muciniphila* in the gut, and ADT reversed this effect. Furthermore, housing PCa-bearing mice with tumor-free mice or administering *Akkermansia* orally enhanced the effectiveness of ADT. This is relevant for individuals with PCa as prolonged ADT leads to higher thymic production, as evidenced by elevated levels of recent thymic emigrant cells in the bloodstream. Healthy volunteer feces successfully restored ADT efficacy, but feces from PCa patients did not have the same effect. These findings suggest the potential clinical utility of reversing intestinal dysbiosis and repairing acquired immune defects in PCa patients (Terrisse et al., 2022a).

## 4 Potential therapies targeting the GM for prostatic disease

**Figure 2:**
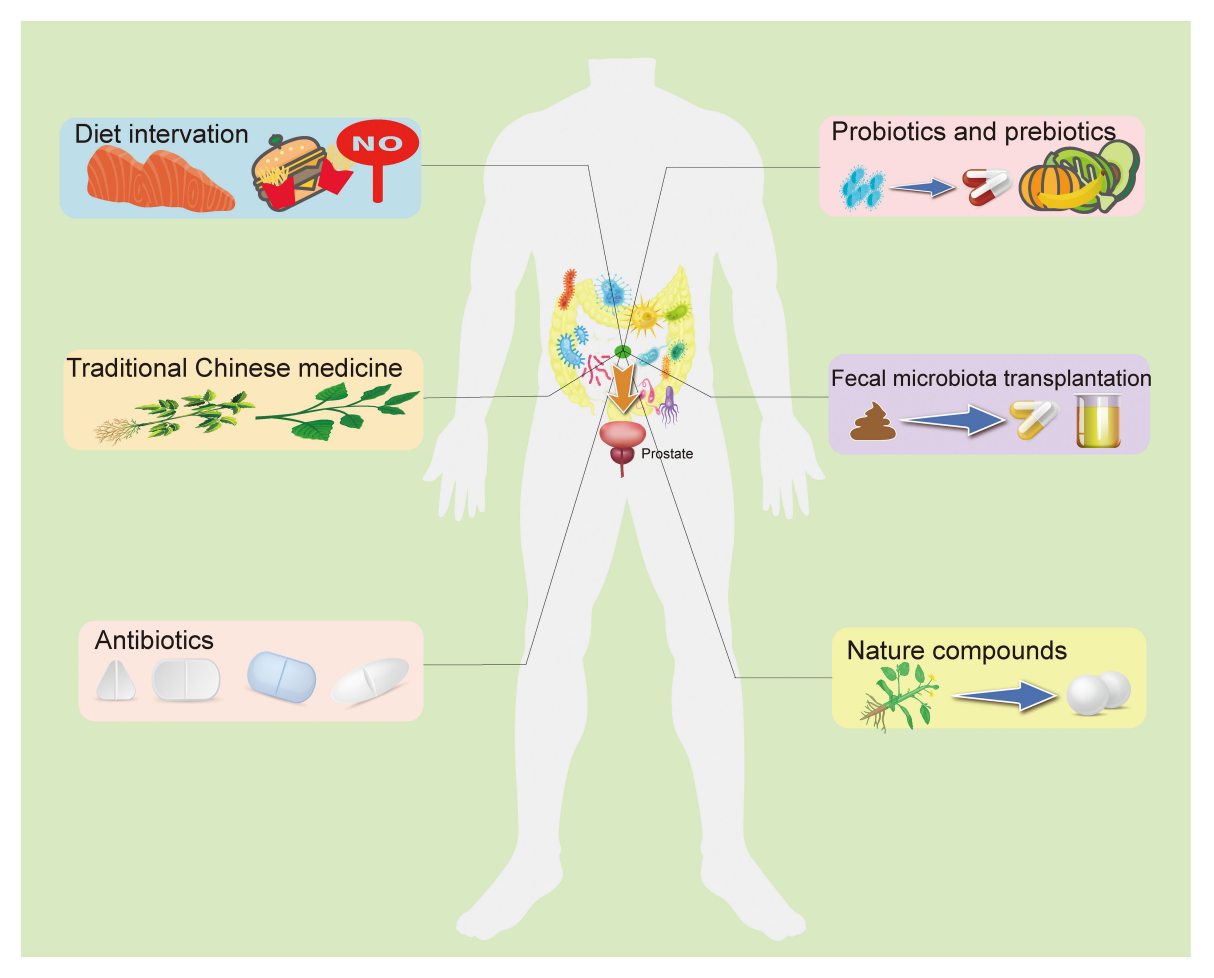
The potential preventive and therapeutic methods target the GM for prostatic disease. The current study suggests that targeting the GM to regulate prostate status is a promising treatment. A high-fat diet is believed to be an important threat to prostatic disease, and fat metabolites from the gut microbiota can influence prostate health in a number of ways, as do other dietary components. Recent research indicated that some TCM used to treat prostatic disease may work by targeting gut microbiota. In addition, antibiotics, probiotics, prebiotics, and fecal bacteria transplantation directly alter the composition of the intestinal flora and the abundance of specific bacteria, improving intestinal disorders. Some natural compounds have been found to interact with the GM to play a role in anti-prostatic disease, and with further research and development, these drug candidates are gradually being understood and applied. GM, Gut microbiome; TCM, Traditional Chinese medicine.

Dysbiosis of the gut microbiota is a result of the imbalance between beneficial and harmful microorganisms. Several factors, including diet, traditional Chinese medicine, antibiotics, probiotics and prebiotics, fecal transplantation, and certain natural compounds, contribute to prostatic disease development and progression by improving this imbalance in the GM, which should be considerable when preventing and treating prostatic disease (Pernigoni et al., 2023) (**Figure 2**).

### 4.1 Diet

Dietary components directly and profoundly impact the GM and diet-derived metabolites. Rational dietary patterns and applying nutritional supplements can help regulate intestinal microbiota homeostasis, thereby preventing and treating prostatic disease. However, the specific diet or supplements that are effective still need to be determined. Recent research has shown that consuming a diet high in fat can increase the risk of developing prostate disease, with the gut microbiota potentially playing a role as a contributing factor (Labbé et al., 2019; Zmora et al., 2019). High-fat diet (HFD) is known to result in an imbalance of gut bacteria and their metabolites, which can contribute to a leaky gut. This allows various metabolites and bacterial components, like SCFAs and phospholipids, to enter the bloodstream, leading to conditions such as endotoxemia. This phenomenon can thus orchestrate the inflammatory response, promoting the initiation and progress of prostatic disease (Zmora et al., 2019).

A rat model of CP/CPPS was created by administering subcutaneous testosterone and 17β-oestradiol (E (2)) hormone pellets, followed by providing either regular tap water or tap water enriched with 2% galactoglucomannan-rich hemicellulose extract (GGM group) from Norway spruce. Rats with hormone-induced CP/CPPS exhibit distinct alterations in gut microbiota, leading to subsequent changes in short-chain fatty acids and lipopolysaccharides (Konkol et al., 2019). Rats with HFD-induced BPH exhibited morphological abnormalities in their prostate tissues. Metagenomic analysis of the gut revealed that *Bacillota*, *Bacteroidota*, and *Ruminococcus spp* exhibited greater abundance in the HFD group. The KEGG analysis revealed that the genes with altered expression were predominantly enriched in pathways such as NOD-like receptor (NLR) signaling, PI3K-Akt signaling, estrogen-signaling, GABAergic synapse-related signaling, and pantothenate and CoA biosynthesis (Gu et al., 2021; Gu et al., 2024).

A study recruited 40 PCa patients who were grouped into weight-loss diet and control, then test the GM of feces. *Pseudomonadota* were plentiful, and the Gleason sum correlated with *Clostridium* and *Blautia*, as discovered by the team. Moreover, a rise in the intake of red meat compared to the initial level was linked to the presence of *Prevotella* and *Blautia bacteria*. Individuals who raised their consumption of poultry experienced a reduction in the abundance of *Eubacteriales (Clostridiales)* (Frugé et al., 2018). A PCa mouse model raised with HFD also showed that HFD could promote prostate carcinogenesis, and microbiota-mediated equol significantly decreased because of the decreased abundance of equol-producing bacterium *Adlercreutzia*. Thus, the authors hypothesized that HFD might promote PCa by adversely affecting equol-producing bacterium (Liu et al., 2019). Studies indicated that diets high in saturated and monounsaturated fatty acids, such as the Lard diet, may increase the risk of developing and progressing PCa. Additionally, the composition of gut bacteria, specifically *Eubacteriales s* and *Lactobacillales*, was significantly different in mice fed the Lard diet compared to those fed a fish oil diet. Furthermore, the regulation of lipid processing and cholesterol production pathways involves three and seven frequently altered genes in PCa tissues, some of which are associated with the prevalence of the *Lactobacillales order* in the mouse intestinal tract. Thus, SMFA could potentially enhance the advancement of PCa by increasing the presence of certain gut bacteria and upregulating lipogenic genes in PCa (Sato et al., 2022). Evidence suggests that indole-3-carbinol (I3C), a compound found in cruciferous vegetables, could potentially provide protection against PCa. The impact of dietary I3C on the GM of mice may be attributed to its modulatory effect (Wu et al., 2019).

### 4.2 Traditional Chinese medicine

Traditional Chinese medicine (TCM) is a unique medical treatment in China, some of which have demonstrated promising efficacy in treating prostatic disease. Recent research indicates that they are able to function by focusing on the GM. Poria cocos polysaccharides (PPs) is an antiandrogenic drug used to treat BPH and CP/CPPS. A study using rats demonstrated that the metabolites of PPs, 7-keto deoxycholic acid and haloperidol glucuronide, were significantly enriched by *Parabacteroides, Fusicatenibacter, and Parasutterella* after fermentation by human fecal microbiota. These metabolites could potentially act as signal molecules to alleviate CP/CPPS (Yu et al., 2022). When compared to finasteride in treating CP/CPPS, PPs and finasteride both significantly improved the histological damage in the inflamed prostate. Additionally, PPs and finasteride suppressed the synthesis of inflammatory cytokines (TNF-α, IL-2, and IL-8) as well as androgens (dihydrotestosterone and testosterone).16S rDNA sequencing revealed that PPs and finasteride induced unique changes in the composition of the gut microbiota. Additional examination indicated that PPs, as opposed to finasteride, reversed alterations in the intestinal microbiota caused by CP/CPPS, specifically affecting *Oscillospiraceae NK4A214* group, an unidentified bacterium from *Oscillospiraceae, Ruminiclostridium 9*, *Phascolarctobacterium, Coriobacteriaceae UCG-002*, and *Oribacterium*. LDA effect size analysis revealed that PPs recovered the gut microbiota by targeting the *Oscillospiraceae NK4A214* group (Liu et al., 2021a). Additional examination of sex hormones showed that PPs may help reduce CP/CPPS by controlling the levels of testosterone (T), dihydrotestosterone (DTH), and estradiol (E2). PPs may help reduce CNP by controlling the levels of inducible nitric oxide synthase, malonaldehyde, and superoxide dismutase in the inflamed prostate, thereby boosting antioxidant activity (Liu et al., 2020).

Rapeseed bee pollen is considered an important remedy for CP/CPPS, as well as having the ability to regulate gut microbiota and enhance intestinal health. Rapeseed bee pollen has the ability to reduce symptoms of CP/CPPS by specifically controlling the gut microbiota, with increased amounts and broken cell wall pollen demonstrating better results. Administering a large amount of rapeseed bee pollen with disrupted walls resulted in a decrease of around 32% in prostate wet weight and 36% in prostate index. Treatment with rapeseed bee pollen that had been disrupted by the wall also led to a significant decrease in the levels of proinflammatory cytokines, including IL-6, IL-8, IL-1β, and TNF-α. Additionally, rapeseed bee pollen has the ability to suppress harmful bacteria and improve beneficial bacteria, especially in the ratio of *Bacillota* to *Bacteroidota* and the quantity of *Prevotella* (genus) (Qiao et al., 2023).

Traditionally, it is a common practice in medicine to use Epilobium sp. for treating the initial phases of BPH and inflammation. Certain components from the extracts (endothelin B, quercetin-3-O-glucuronide, myricetin-3-O-rhamnoside) demonstrated activity in LNCaP cells. Further analysis revealed that ellagitannins from Epilobium hirsutum herbs (a kind of Epilobium sp.) Human gut microbiota was shown to convert extracts into urolithins. Urolithin C exhibited strong effects in suppressing cell growth, PSA release, and arginase function (Stolarczyk et al., 2013; Piwowarski et al., 2017).

### 4.3 Antibiotics

Antibiotics are seen as a key factor in causing dysbiosis in the gut microbiota, and short-term antibiotic exposure for patients undergoing prostate biopsy skews the GM composition, leading to long-lasting changes in the gut microbiota that are difficult to reverse (Li JKM. et al., 2022; Tóth et al., 2022). Antibiotics are primarily utilized for treating upper respiratory tract infections (URIs) and urinary tract infections (UTIs), particularly in elderly males who often experience complications such as PCa and BPH in clinical settings (Ong et al., 2008). In Korea, a retrospective cohort study based on the population found that over one million people who used antibiotics for 180 days or longer had an increased risk of PCa compared to those who did not use antibiotics. Furthermore, those who utilized four or more types of antibiotics were at an increased risk of PCa compared to those who did not use antibiotics (Park et al., 2023). Research examined the link between antibiotic usage rates and cancer susceptibility across 30 European nations, finding that countries with elevated consumption of specific types of penicillin and tetracycline had a greater likelihood of developing PCa (Ternák et al., 2020). Weibo Zhong’s study found that disturbing the GM with broad-spectrum antibiotics in water led to the development of subcutaneous and orthotopic tumors in mice. Subsequent analysis of mouse feces using 16S rRNA sequencing revealed a notable increase in *Pseudomonadota* levels following antibiotic treatment, leading to heightened gut permeability and intertumoral LPS, ultimately facilitating the progression of PCa through the NF-κB-IL6-STAT3 pathway in mice (Zhong et al., 2022). Given that antibiotics have such a strong ability to regulate the GM, it is also essential to consider how structural changes in the microbiota might affect the treatment of disease when using antibiotics in the clinic.

### 4.4 Probiotics and prebiotics

Probiotics, which are live microorganisms that promote health, primarily consist of *Bifidobacterium* and *Lactiplantibacillus* strains, along with some *Enterococcus* and *Streptococcus* strains (Wilkins and Sequoia, 2017). The presence of beneficial bacteria in PCa patients, like *Prevotella* spp., has been linked to higher rates of patient survival. Live probiotics could potentially be utilized to postpone the development of aggressive PCa, as shown in previous studies on preclinical mice (Pernigoni et al., 2021). Changes in the gut microbiota of the host can impact multiple organisms, suggesting that a carefully planned group of bacteria with diverse effects from various strains could be more effective than giving just one type of live bacteria as a probiotic. A potential combination of bacteria for treating PCa could consist of various species of *Prevotella*. They are positively correlating with patients’ survival (Pernigoni et al., 2023). Moreover, androgen catabolic intestinal bacteria may be helpful as a potent biogenic organism in hyper-androgen replacement therapy.

Prebiotics are indigestible components of food that have a positive impact on human health by promoting the growth of certain beneficial gut bacteria. Studies have shown that prebiotics can impact immune regulation, microbial protection, as well as bowel and metabolic processes (Sanders et al., 2019). In a study involving 30 patients with endometrial, cervical, colon, rectal, or PCa undergoing pelvic radiotherapy, a randomized, double-blind controlled trial was conducted. The patients were either given partially hydrolyzed guar gum or a placebo. The results indicated a decrease in diarrhea frequency and an increase in *Bifidobacterium* count in the group that received partially hydrolyzed guar gum. However, there was no notable variation in quality-of-life scores (Rosli et al., 2021). Therefore, utilizing routines that support healthy gut microbes with probiotics/prebiotics could reduce the likelihood of prostate cancer development in high-risk men.

### 4.5 Fecal microbiota transplantation

FMT, a recent technique, involves transferring fecal material from a healthy donor into the recipient’s intestines to balance microbial populations. FMT can be administered in fresh or frozen samples, as well as in capsule form, through either an upper or lower gastrointestinal route. Some research indicates that the lower gastrointestinal route may be more effective for this purpose, possibly due to the presence of colonic pathology (Baunwall et al., 2020; Walter and Shanahan, 2023). Research conducted on FMT using stool samples from healthy donor mice or HSPC patients has demonstrated a delay in the development of castration resistance when compared to FMT from CRPC mice or patient donors (Pernigoni et al., 2021). The combined research suggests that FMT could be a viable approach to transferring beneficial bacteria from those who respond well to those who do not, in order to address resistance to treatment. Weimin Dong et al. discovered that ulcerative colitis led to an enlargement of the prostate, with elevated levels of GPER expression that could be reversed by FMT. Furthermore, prostate tissues exhibited higher butyric acid levels after they were treated with FMT. *In vitro* experiments showed that the fecal filtrate (FF) from healthy mice increased the expression of GPER, suppressed cell proliferation, and triggered apoptosis in BPH-1 cells (Dong et al., 2022). A previous study showed that *Ruminococcus* correlated with phospholipid metabolism was more abundant in CRPC than in HSPC individuals. Treatment with CRPC feces hastened the progression of prostate cancer in mice and elevated the levels of *Ruminococcus* in their intestines. CRPC FMT treated mice showed increased levels of most fecal lipids, such as lysophosphatidylcholine and phosphatidylcholine, along with higher levels of LPCAT1, RAD51, and DNA-PKcs in the prostate, indicating that *Ruminococcus* may promote PCa progression by upregulating LPCAT1 and DNA repair proteins (Liu Y. et al., 2021). The results showed that fecal transplantation with certain beneficial bacteria is a crucial question that needs consideration, and future research to explore beneficial ones is essential.

### 4.6 Natural compound

In addition to the standard therapies that target the gut microbiota mentioned above, some natural compound candidates have shown promising therapeutic potential. Astaxanthin is a naturally occurring substance that possesses anti-inflammatory and immunomodulatory effects, as well as probiotic properties. AST administered orally led to an increase in the proportion of *Akkermansia muciniphila*, resulting in higher levels of SCFAs acetate in the blood, enhanced expression of colonic tight junction markers, reduced levels of serum lipopolysaccharide, ultimately reducing inflammation and pain in EAP mice (Liu et al., 2024). The extract from blackberry seeds boosted the overall quantity of intestinal bacteria. It altered the prevalence of particular bacteria and demonstrated abilities for reducing inflammation and inhibiting cell growth by suppressing the expression of IL-1β mRNA induced by LPS in the proliferation of LNCaP cells (Choe et al., 2020). Phytoestrogens called lignans, obtained from a variety of plants, can be metabolized by the gut microbiota in humans to produce enterodiol (END) or enterolactone (ENL). Lignans have been identified for their antioxidant and anti-inflammatory effects, as well as their involvement in estrogen receptor-related pathways. Additionally, lignans have shown effectiveness in inhibiting tumor growth in different types of cancer cells, including PCa. The molecular pathways of lignans in treating PCa include the suppression of inflammatory signals, such as the NF-κB pathway (Rowland et al., 2003; Hålldin et al., 2019; Jang et al., 2022). Polyphenols from berries are active compounds produced and released by various types of berry fruits. These substances hinder the growth of harmful bacteria and support helpful bacteria, which helps reduce inflammation by blocking Nf-κB and preventing the development of PCa. Research findings indicated that polyphenols found in berries have the potential to be a valuable source of bioactive substances that can influence the GM and help in the treatment of PCa (Bouyahya et al., 2022). Green tea catechins (GTCs) could influence the molecular pathways involved in the development of PCa. Orally administering the GM enzyme changes the structure of GTC, impacting its availability, activity, and toxicity while also controlling inflammation and hormone pathways (Stanisławska et al., 2019; Kumar et al., 2022). Pomegranate juice contains ellagitannins (ETs), which are polyphenols that have the potential to prevent PCa through their bioactive properties. Extraterrestrials undergo partial hydrolysis in the digestive system, transforming into ellagic acid (EA) and further into Uro A. These compounds travel through the bloodstream to various parts of the body, including the prostate, where they inhibit the proliferation of prostate cancer cells and stimulate their programmed cell death (Seeram et al., 2007; González-Sarrías et al., 2010; Vicinanza et al., 2013). As more drug candidates become available, approaches for regulating the microbiome will diversify, and it will be critical to explore whether combined regulation between these drugs can enhance or influence each other.

In conclusion, current observational and experimental data suggest that some gut microbiotas differ between prostatic disease patients and healthy people. Additionally, basic experiments indicate that the GM can affect prostate health through a variety of mechanisms, including direct pathogen invasion, immune regulation, metabolite and androgen regulation, and so on. This evidence suggested that targeting the gut microbiota in the treatment of prostatic disease may have a positive effect. The initial studies of the aforementioned treatments have confirmed this idea, but these treatments have not yet been used in the clinic. In the future, we will need to conduct more experiments to confirm the possibility of targeting the GM to treat prostate disease.

## 5 Conclusion and future directions

There is a small but growing body of work investigating the GM and its relationship with prostatic health and disease. This review provides an overview of the observational and animal data that demonstrate the relationship between GM and prostatic disease. Then, we specifically analyze the potential mechanisms mediating this phenomenon, involving direct infection, inflammation, immunity, metabolites derived from the GM, androgens, and ADT. With this in mind, we propose six potential ways to target GM to prevent or treat prostatic disease, including diet intervention, TCM, antibiotics, probiotics and prebiotics, FMT, and natural compounds. Overall, targeting the GM to maintain prostate health is a promising approach. However, it should be noted that current research into the GM and prostate is limited in certain respects. Initially, the current study suggests possible pathogenesis, but the mechanisms do not fully explain the occurrence of prostate disease. How important the role of the GM in the pathogenesis of prostate disease is still unclear, as is the extent of benefit that can be obtained from targeted therapy. Furthermore, previous studies on the mechanism are based on animal studies, and whether it exists in humans is still unknown. In addition, the question of whether these treatments have side effects and whether the combination of these potential treatments can achieve greater efficacy is also worth further investigation. Last, in light of the diverse range of prostate diseases affecting patients, it is crucial to investigate further the efficacy of various treatment methods. We hope to have more research to reveal the specific gut microbiota and its effect mechanism, in order to develop more precise treatment to protect prostate health.

## Author contributions

HC: Writing – original draft, Writing – review & editing. DZ: Writing – original draft. PW: Writing – original draft. YW: Writing – original draft, Writing – review & editing. CS: Writing – original draft. HW: Writing – original draft. HD: Writing – original draft. WZ: Writing – original draft. ZG: Writing – original draft. HZ: Writing – review & editing. SW: Writing – review & editing.
